# The double-edged roles of ROS in cancer prevention and therapy

**DOI:** 10.7150/thno.56747

**Published:** 2021-03-04

**Authors:** Yawei Wang, Huan Qi, Yu Liu, Chao Duan, Xiaolong Liu, Tian Xia, Di Chen, Hai-long Piao, Hong-Xu Liu

**Affiliations:** 1 Department of Thoracic Surgery, Cancer Hospital of China Medical University, Liaoning Cancer Hospital & Institute, Shenyang 110042, China.; 2 CAS Key Laboratory of Separation Science for Analytical Chemistry, Dalian Institute of Chemical Physics, Chinese Academy of Sciences, Dalian 116023, China.; 3 Department of Biochemistry & Molecular Biology, School of Life Sciences, China Medical University, Shenyang, 110122, China.

## Abstract

Reactive oxygen species (ROS) serve as cell signaling molecules generated in oxidative metabolism and are associated with a number of human diseases. The reprogramming of redox metabolism induces abnormal accumulation of ROS in cancer cells. It has been widely accepted that ROS play opposite roles in tumor growth, metastasis and apoptosis according to their different distributions, concentrations and durations in specific subcellular structures. These double-edged roles in cancer progression include the ROS-dependent malignant transformation and the oxidative stress-induced cell death. In this review, we summarize the notable literatures on ROS generation and scavenging, and discuss the related signal transduction networks and corresponding anticancer therapies. There is no doubt that an improved understanding of the sophisticated mechanism of redox biology is imperative to conquer cancer.

## Background

Reactive oxygen species (ROS) are two electron reduction products of oxygen, including superoxide anion, hydrogen peroxide, hydroxyl radical, lipid peroxides, protein peroxides and peroxides formed in nucleic acids 1. They are maintained in a dynamic balance by a series of reduction-oxidation (redox) reactions in biological systems and act as signaling molecules to drive cellular regulatory pathways 2, 3. Excessive oxidative stress derived from ROS accumulation deregulates the antioxidative defense system, which is closely associated with various diseases 4, 5, especially cancers 6. Though emerging studies have demonstrated the primary ligand stimulants, the enzymatic generation mechanisms as well as the putative downstream targets 6, 7, the major mechanisms by which ROS participates in cancer development in concentration-dependent, spatially dependent and temporally dependent manners remain insufficiently understood.

During different stages of cancer formation, abnormal ROS levels play paradoxical roles in cell growth and death 8. A physiological concentration of ROS that maintained in equilibrium is necessary for normal cell survival. Ectopic ROS accumulation promotes cell proliferation and consequently induces malignant transformation of normal cells by initiating pathological conversion of physiological signaling networks. Excessive ROS levels lead to cell death by damaging cellular components, including proteins, lipid bilayers, and chromosomes. Therefore, both scavenging abnormally elevated ROS to prevent early neoplasia and facilitating ROS production to specifically kill cancer cells are promising anticancer therapeutic strategies, in spite of their contradictoriness and complexity. Consequently, a better understanding of the sophisticated mechanism of ROS in tumorigenesis is critical to conquering cancer.

In this review, we not only discuss the double-edged roles and molecular regulatory mechanisms of ROS in cancer prevention and therapy, but also summarize the relevant small molecule interventions and envision the future perspectives of ROS-targeted cancer treatment.

## Sources of ROS

Approximately 2.4-3.8 billion years ago, ROS most likely appeared on the earth along with the first oxygen molecule in the atmosphere, and they have existed with the aerobic life ever since 9, 10. In human cells, biologically relevant ROS are derived from both the exogenous environment and endogenous metabolism.

### Exogenous ROS generation

Exogenous ROS can be generated from exposure to air pollutants, tobacco, metals, asbestos or radiation. From the perspective of air pollutants, a recent study has shown that exposure to fine particulate matter with a diameter of less than 2.5 µm (PM 2.5) can lead to DNA damage in bronchial epithelial cells 16HBE by inducing oxidative stress 11. Regarding tobacco, detrimental components generated from tobacco smoking can induce oxidative stress by decreasing the circulating concentrations of antioxidant micronutrients such as cryptoxanthin, α carotene, β carotene, and ascorbic acid 12. As a class I carcinogenic heavy metal, arsenic can increase ROS levels via Fenton reaction, thus participating in the progressions of multiple malignancies 13, 14. Inhaled asbestos fibers accumulate in lungs and induce the generation of ROS due to the presence of iron associated with the fibrous silicates, which promote the malignant transformation of mesothelial cells 15. Ultraviolet radiation (UVR) increases oxidative stress not only by upregulating nitric oxide synthase (NOS) synthesis but also by impeding catalase (CAT) to scavenge hydrogen peroxide, thus leading to increased risk of sunburn, photoaging and skin cancer 16. Ionizing radiation (IR) stimulates ROS generation by immediately inducing extracellular water radiolysis or causing intracellular mitochondrial metabolic disorder, thereby destroying cancer cells or, conversely, facilitating their survival and metastasis 17. Besides, many carcinogens in the environment play oncogenic roles by inducing ROS accumulation.

### Endogenous ROS generation

**Figure 1:**
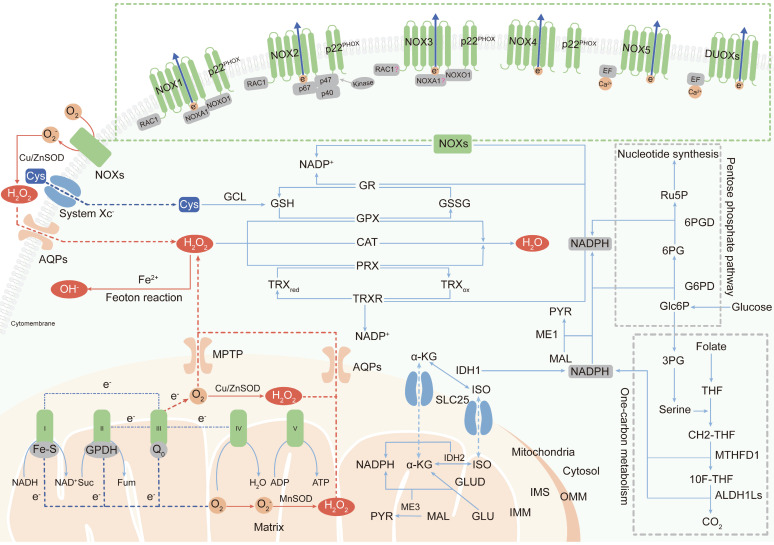
** The primary generation and elimination mechanisms of intracellular ROS.** (Ⅰ) The two major sources of endogenous ROS are mitochondrial ETC and NOXs: oxygen gains electrons leaked from complex Ⅰ, Ⅱ and Ⅲ of ETC to generate superoxide; NOXs transfer electrons from NADPH in the cytosol to oxygen at extracellular to produce superoxide; superoxide is dismutated to H_2_O_2_ by SOD for subsequent transmembrane transportation. (Ⅱ) Intracellular ROS are scavenged by three enzymatic antioxidants: PRXs, GPXs and CAT. PRXs and GPXs use NADPH as reducing equivalent; CAT use non-NADPH hydrogen donors. (Ⅲ) NADPH is synthesized in cytoplasm and mitochondria: cytosolic NADPH is primarily generated from PPP pathway; in mitochondria, GLUD generate NADPH by converting glutamate to α-KG; IDHs and MEs are contributed to NADPH pools both in the cytosol and mitochondrial by respectively catalyzing the oxidative decarboxylation of isocitrate to α-KG and malate to pyruvate; one-carbon metabolism contributes to NADPH production both in cytosol and in mitochondria.

The two major sources of endogenous ROS are the mitochondrial respiratory chain, which generates ROS as a byproduct 18, 19, and active NADPH oxidases (NOXs), whose primary function is ROS production. In addition, peroxisomes and endoplasmic reticulum membranes have also been identified as cellular sites of ROS generation 20, 21. Here, we mainly discuss research status on ROS production in mitochondria and by NOXs (Figure 1).

#### Mitochondria-derived ROS

In mammalian cells, the mitochondrial electron transport chain (ETC) is the main source of ATP 22. However, during oxidative phosphorylation and energy transduction, approximately 1% of molecular oxygen gains electrons leaked from the ETC, yielding superoxide 23. Some superoxide is released into the cytoplasm through the mitochondrial permeability transition pore (MPTP) located in the outer mitochondrial membrane (OMM) 24. However, most superoxide is dismutated to H_2_O_2_ by superoxide dismutases (SODs) in the mitochondrial matrix or intermembrane space (IMS) 25. H_2_O_2_ is highly diffusible and specifically carried into the cytoplasm by aquaporins (aquaporins 3 and 8) as a second messenger to regulate multiple signaling pathways 26, 27.

The mammalian mitochondrial respiratory chain mainly contains five complexes including NADH-ubiquinone oxidoreductase (complex I), succinate-ubiquinone oxidoreductase (complex II), cytochrome bc_1_ complex (complex III), cytochrome c oxidase (complex IV), and ATP synthase (complex V) 28. It is believed that complexes I and III are the main sites of superoxide generation 29. The iron-sulfur cluster (Fe-S) in the matrix-protruding hydrophilic arm in complex I is the potential site of electron leakage, and the generated superoxide is exclusively discharged into the mitochondrial matrix 30, 31. The ubiquinol oxidation site (Q_o_ site) is the locus of superoxide production in complex III and releases superoxide to both sides of the inner mitochondrial membrane (IMM) 32. No more than 50% of the superoxide produced from electron leakage at complex III is excreted to the intermembrane space; the remaining 50% is released into the mitochondrial matrix 31. In addition to complexes I and III, a fraction of the superoxide production in mitochondria is attributed to flavin-reducing site (II_F_) within complex II 33.

Although the initial anionic form of superoxide generated via the ETC is too strongly charged to readily cross the inner mitochondrial membrane, instantaneously conversion into H_2_O_2_ by SODs permits the transportation of ROS from mitochondria to cytoplasm 34. However, it has also been reported that a fraction of superoxide released by complex III in the intermembrane space can diffuse directly into the cytoplasm through the MPTP 35. Metabolic equilibrium of mitochondrial ROS is important in processes promoting the normal function of cells, including Ca^2+^ homeostasis and ATP synthesis 36, 37. Under aberrant physiopathological conditions, as a casualty, mitochondrial DNA damage and component dysfunction induced by ROS accumulation lead to malignant transformation 38.

#### NOX-derived ROS

The transmembrane NADPH oxidases are identified as the only enzyme family with the sole role of generating ROS and are first described to show bactericidal activity in phagocytes by yielding superoxide 39. Later studies identified the other six cytochrome homolog subunits including NOX1, NOX3-5, dual oxidase 1 (DUOX1) and dual oxidase 2 (DUOX2) 40. Together with the phagocyte NADPH oxidase (NOX2/gp91^PHOX^), these homologs are now referred to as the NOX family, and all of them have the ability to transport electrons across the plasma membrane and produce ROS 41. All NOXs share similar structures including six transmembrane domains with two heme-binding regions and an NADPH-binding region in the intracellular C-terminus, but their organ distribution and regulatory mechanisms are different 42.

NOX2 is a prototypical NOX family member and first identified as cytochrome b558, which is absent in the bactericidal-deficient leukocytes of chronic granulomatous disease patients 43, 44. NOX2 is widely distributed in various tissues and is composed of the protein scaffold gp91^PHOX^, the membrane partner p22^PHOX^, the GTP-binding protein RAC1 and three cytosolic subunits (p67^PHOX^, p47^PHOX^ and p40^PHOX^) 45. Phosphorylated p47^PHOX^ acts as a “launching switch” to interact with p22^PHOX^ and organizes the translocation of the catalytic subunits (p67^PHOX^, p40^PHOX^) and the energy-providing subunit (RAC1) to the complex. Once assembled, NOX2 is activated and transfers electrons from NADPH in cytosol to oxygen in extracellular space to produce superoxide 46.

NOX1 is the first discovered isoform of NOX2 and is highly expressed in colonic epithelium 47, 48. NOX organizer 1 (NOXO1, a homolog of p47^PHOX^) and NOX activator 1 (NOXA1, a homolog of p67^PHOX^) are novel cytosolic subunits of NOX1. Besides, NOX1 activation also requires the membrane partner p22^PHOX^ and the GTPase Rac1 49.

NOX3 is defined by two seminal contributions which reveal that NOX3 mutations give rise to vestibular defects in head tilt mice 50, 51. p22^PHOX^ and NOXO1 are essential partners for NOX3 activation 52, 53. Whether the other cytosolic subunits, such as NOXA1, p47^PHOX^, p67^PHOX^ and RAC1, are also involved in NOX3 assembly is still controversial.

NOX4 is initially identified as a NADPH oxidase in the kidney 54, 55. In contrast to NOX1-3 activation, NOX4 activation is independent of the cytosolic subunit 56, and the subunit required for NOX4-derived ROS production is p22^PHOX^ 57. A question about NOX4 is its uncertain subcellular localization. As a transmembrane protein, NOX4 has been found to be localized at the cytomembrane and endoplasmic reticulum (ER), but NOX4 expression has also been observed in the nucleus 58, 59, which is difficult to understand how a membrane-spanning protein can be located in a presumably membrane-free space.

NOX5 is a unique and unusual NADPH oxidase. Its structure is distinguished from the NOX1-4 homologs by the presence of a long intracellular NH2 terminus containing a Ca^2+^-binding EF-hand domain and it does not require membrane partner, cytosolic subunit or GTPase for activation 60. The only activator of superoxide generation by NOX5 is the intracellular Ca^2+^ concentration, which is documented by its inactivated function of ROS production under Ca^2+^-free conditions 61. To date, only the NOX5 crystal structure has been discovered (in 2017; the first and only NOX crystal structure to be solved) 62.

DUOX proteins, also known as thyroid oxidases, were first defined as NADPH oxidases in thyroid epithelial cells 63, 64. DUOXs can be directly activated by Ca^2+^ and do not require cytosolic activator or organizer subunits, suggesting that their EF-hand Ca^2+^-binding domains are functional 65. Although DUOXs are homologous with peroxidases, it is not clear whether they can function as peroxidases 66.

The superoxide produced by NOXs in extracellular is transported into the cytoplasm in the form of H_2_O_2_ 67, which acts as a secondary messenger to maintain cell survival and proliferation or, paradoxically, to induce cell death 68, 69. Abundant researches have shown that ROS accumulation induced by over-activation of NOXs contribute to both the initiation and the progression of malignancies. For example, NOX-2 derived superoxide drives mitochondrial to transfer from bone marrow stromal cells to leukemic blasts^70^. NOX1-generated ROS promotes the self-renewal activity of CD133^+^ thyroid cancer cells through activation of the Akt signaling 71. NOX4-driven ROS formation regulates proliferation and apoptosis of gastric cancer cells through the GLI1 pathway 72. Therefore, a deep understanding of the precise roles of NOXs will pave the way for their validation as anticancer therapeutic targets.

## Antioxidant defense mechanisms

Antioxidant defense systems sustain the balance between the generation and neutralization of ROS to maintain redox equilibrium and protect macromolecules from indiscriminate destruction inflicted by oxidative stress (Figure 1).

### Intracellular enzymatic antioxidants

Superoxide dismutases (SODs), categorized as cytosolic SOD1, mitochondrial SOD2, and extracellular SOD3, are widely distributed in various cellular compartments and can rapidly dismutate superoxide into H_2_O_2_ 73. Then, peroxiredoxin (PRX), glutathione peroxidase (GPX) and catalase (CAT) convert H_2_O_2_ into water (H_2_O). The primary sites in PRXs, GPXs and CAT that react with H_2_O_2_ are cysteine, selenocysteine and heme, respectively 74-76. Among these enzymes, PRXs are thought to be ideal H_2_O_2_ scavengers, given their high-affinity binding sites for H_2_O_2_ and their abundant expressions and broad distributions in subcellular compartments 77. Oxidized PRXs are reduced by thioredoxin (TRX). TRX is then restored to their reduced form by thioredoxin reductase (TRXR) and the reducing equivalent provided by NADPH 77. GPXs convert H_2_O_2_ to H_2_O by oxidizing reduced glutathione (GSH) to glutathione disulfide (GSSG), and glutathione reductase (GR) reduces oxidized GSSG back to GSH using NADPH as an electron donor 78. Unlike PRXs and GPXs, CAT converts H_2_O_2_ to H_2_O without the participation of any cofactors. CAT participates in two different antioxidant reactions according to the H_2_O_2_ concentration. At high H_2_O_2_ levels, CAT exhibits catalytic activity by converting H_2_O_2_ into H_2_O and O_2_. At low H_2_O_2_ levels, CAT exhibits peroxidatic activity to reduce one H_2_O_2_ molecule into two H_2_O molecules by consuming two reducing equivalents from non-NADPH hydrogen donors, such as alcohols, phenols, hormones and metals 79.

### NADPH, the primary intracellular hydrogen donor

NADPH, as the primary hydrogen donor and component of the reducing equivalent pool, maintains moderate disulfide reduction of most proteins through NADPH-dependent TRXR and GR redox systems 80. Maintaining the dynamic equilibrium of the compartmentalized NADP+/NADPH pools is essential for cellular redox homeostasis and modulation of thiol-disulfide transformation signaling 81.

NADPH is generated via various metabolic processes in the cytosol or mitochondria. Cytosolic NADPH is primarily generated via the pentose phosphate pathway (PPP) by glucose-6-phosphate dehydrogenase (G6PD) and 6-phosphogluconate dehydrogenase (6PGD) 82. In addition, isocitrate dehydrogenases (IDHs) and malic enzymes (MEs) also contribute to the NADPH pools in the cytosol and mitochondria. IDHs and MEs catalyze the oxidative decarboxylation of isocitrate and malate to α-ketoglutarate (α-KG) and pyruvate, respectively 83, 84. In mitochondria, glutamate dehydrogenases (GLUDs) can generate NADPH by converting glutamate (GLU) to α-KG 85. In addition to the conventional knowledge described above, serine-driven one-carbon metabolism can also contribute to the NADPH production according to recent studies, in which the oxidation of methylene tetrahydrofolate (CH2-THF) to 10-formyl-tetrahydrofolate (10F-THF) by MTHFD1 and the conversion of 10F-THF to CO_2_ by ALDH1Ls (ALDH1L1 in cytosol and ALDH1L2 in mitochondria, respectively) are both accompanied by the reduction of NADP^+^ to NADPH 86, 87.

Because the IMM is impermeable to NADPH, NADPH communication between the cytosol and mitochondria is conducted via the isocitrate-a-KG shuttle through the mitochondrial transporter SLC25 88. In mitochondria, IDH2 converts α-KG to isocitrate using NADPH. Then, isocitrate turns into citrate and is transported to cytosol via SLC25. The cytosolic enzyme IDH1, finally, catalyzes the oxidative decarboxylation of isocitrate to α-ketoglutarate and produces NADPH 89.

Superoxide and hydrogen peroxide are the partially reduced forms of oxygen, as it is fully reduced to H_2_O with the addition of four electrons. NADPH, as the electron carrier, is not only a producer (incomplete reduction) but also a scavenger (complete reduction) of ROS. Paradoxically, inadequate NADPH leads to ROS accumulation, and excessive NADPH leads to reductive stress, in particular, being utilized by NOXs to produce ROS. Therefore, only if the NADP+/NADPH level remains in an equilibrium state, NADPH can perform its antioxidant defensive roles.

### NRF2, the master regulator of the antioxidant defense system

Nuclear factor E2-related factor 2 (NRF2), an intracellular transcription factor, controls the expression of antioxidant genes and protects cells from oxidative and electrophilic stress 90. NRF2 is a leucine zipper member of the cap 'n' collar (CNC) family and is composed of six highly conserved Nrf2-ECH (Neh) domains 91. Neh family proteins (Neh1-Neh6) contain a CNC-type leucine zipper domain, which is crucial for DNA binding and dimerization with other transcription factors, such as CREB-binding protein (CBP) and brahma-related gene 1 (BRG1) 92. Kelch-like ECH-associated protein 1 (KEAP1) triggers proteasomal degradation of Nrf2 via CUL3-dependent E3-ubiquitin ligase-mediated ubiquitination 93, 94. When intracellular ROS are accumulated abnormally, the cysteine residues in KEAP1 are oxidized, thus blocking NRF2 interaction and the subsequent degradation 95. Then, the stabilized NRF2 protein is translocated into the nucleus to bind with antioxidant response elements (AREs) and activate the transcription of enzymatic antioxidants such as CAT, PRX and GPX, as well as enzymes involved in GSH metabolism 96. Furthermore, it has also been demonstrated that NRF2 activation increases the production of NADPH by transcriptionally activating the key enzymes in the PPP and one-carbon metabolism pathways 97, 98.

## ROS, a double-edged sword in cancer progression

The double-edged roles of ROS in cancer progression are complicated. We hypothesize this contradiction is rooted in the fact that ROS do not operate as a single biochemical entity but play diverse roles as secondary messengers in a manner defined by their variable concentrations, distributions and durations.

### The carcinogenic effects of ROS

Beginning in the 1990s, studies set the stage for the concept that ROS are a driving factor for tumorigenesis 99. One important characteristic of cancer cells is their increased ROS levels compared to those in their counterpart cells and the subsequently elevated levels of antioxidants to detoxify the accumulated ROS to reinstitute a redox balance. ROS are thought to play oncogenic roles by contributing to activation of proto-oncogenes and inactivation of tumor suppressor genes and by acting as signaling molecules to induce abnormal cell growth and metastasis.

#### ROS-related carcinogenic genetic alterations

Excessive ROS generated by environmental carcinogens, mitochondrial ETC or NADPH oxidases induce DNA damage, including depurination and depyrimidination, single- and double-stranded DNA breaks, base modifications and DNA-protein crosslinks 100. Moreover, ROS not only delay the identification of damaged regions by affecting sensor kinases (ATM and ATR) and downstream transducer kinases (CHK1 and CHK2) 101, 102, but also impair the activation of DNA repair enzyme OGG1 by oxidation of critical cysteine residues 103. Accumulation of DNA lesions affects the interpretation and transmission of genetic information, which leads to permanent changes in genetic material and is one of the vital steps involved in carcinogenic mutagenesis and tumor transformation 104. 8-Hydroxy-2 deoxyguanosine (8-oxo-dG), an oxidizing adduct generated by ROS-associated DNA damage, is commonly utilized to test intracellular oxidative stress levels and is highly expressed in a variety of malignant tumor tissues than in the matched normal ones 105, 106.

Besides the direct carcinogenic effects of DNA damage and chromosomal instability, ROS also act as signaling molecules regulated by oncogene activation or anti-oncogene inactivation. Ectopic expression of the proto-oncogene p21^RAS^ in NIH3T3 fibroblast cells produces large amounts of superoxide via RAC1 activation in NOX complexes and leads to enhanced mitogenic activity, which can be reversed by the chemical antioxidant N-acetyl-L-cysteine (NAC) 107. Trp53-knockout mice, which succumb to neoplasia at the age of 6 months, exhibit elevated ROS levels and karyotypic abnormalities in different organs. Strikingly, prepartum administration of NAC-supplemented water and continuation of this treatment throughout the life of Trp53-/- progeny can extend the lifespan and suppress lymphoma formation 108. The breast cancer susceptibility gene, BRCA1, can protect the cells under oxidative stress. Embryonic fibroblasts with genetic ablation of BRCA1 show higher ROS levels than those from wild-type mice 109. In contrast, BRCA1 overexpression in breast cancer cells stimulates ARE-driven transcriptional activity and upregulates phase II antioxidant enzymes, including glutathione S-transferase and glutathione peroxidase, by enhancing the activity of NRF2 110.

#### ROS driven cellular proliferation

**Figure 2:**
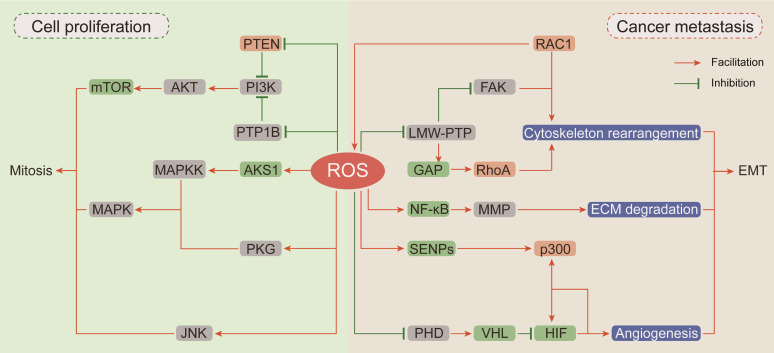
** The carcinogenic effects of ROS.** (Ⅰ) ROS drive proliferation by activating of PI3K/AKT/mTOR and MAPK mitogenic signaling cascades: the devitalized oxidation of PTEN and PTP1B impair their inhibition on PI3K and cause the hyper-activation of AKT and mTOR; ROS accumulation can respectively activate ASK1, PKG and JNK to further stimulate the downstream MAPKK and MAPK mitosis cascades. (Ⅱ) ROS participate in cancer cell EMT: RAC1 not only directly affects cytoskeleton rearrangement but also up-regulates FAK or inhibits RhoA expression through ROS generation to promote cytoskeleton rearrangement; ROS pile up increases MMP expression by activating NF-κB phosphorylation to enhance ECM degradation; ROS suppress HIF ubiquitin degradation and promote its interaction with p300 to induce angiogenesis.

In recent decades, researches have been focused on ROS-dependent stimulation of cellular proliferation. Among them, the seminal achievements are the identification of ROS as second messengers participating in growth factor activation via the PI3K/AKT/mTOR and MAPK/ERK mitogenic signaling cascades (Figure 2).

Thiol-disulfide transformation upon H_2_O_2_ treatment leads to reversible inactivation of phosphatase and tensin homolog (PTEN) 111, which prompted researchers to investigate the mechanism of ROS in neoplastic progression. Then, oxidation-dependent conversion of cysteine into sulfenyl amide in the catalytic subunit of protein tyrosine phosphatase (PTP) 1B was described, and this conversion is consequently accompanied by a conformational change and inhibition of substrate binding 112. Both PTEN and PTP1B are negative regulators of phosphoinositide 3-kinase (PI3K) and protein kinase B (AKT) 113. Given the importance of PI3K/AKT in mitogenic signaling cascades, hyperactivation of this pathway by upstream devitalized oxidation of PTEN/PTP1B is a hallmark of malignancies 114, 115. In neuroblastoma cells, excessive ROS generated by NOXs after insulin stimulation causes oxidative inactivation of PTEN and phosphorylation activation of PI3K/AKT. In neuroblastoma cells pretreated with the NOX inhibitor diphenyleneiodonium (DPI) before insulin stimulation, insulin-induced phosphorylation of PI3K/Akt is markedly reduced 116. In breast cancer cells, transient H_2_O_2_ accumulation via chemokine CXCL12-activated NOX2 results in oxidation of PTEN and PTP1B followed by activation of PI3K/AKT, but the accumulated H_2_O_2_ can be neutralized by DPI and CAT 113.

The mitogen-activated protein kinases (MAPKs) comprise four homologs including extracellular signal-related kinases (Erk1/2), c-Jun N-terminal kinase (JNK), p38 kinase (p38), and big MAP kinase 1 (BMK1/Erk5), and they are activated by a three-rung kinase tier: MAPK kinase kinases (MAPKKKs), MAPK kinases (MAPKKs) and MAPKs 117. Apoptosis signal-regulated kinase 1 (ASK1), an MAPKKK, is inactivated by the reduced form of thioredoxin (TRX) through inhibition of Thr-838 phosphorylation in the activation loop 118. ROS accumulation or antioxidant deficiency induces the oxidation of TRX, resulting in its dissociation from ASK1 and allowing subsequent restoration of ASK1 kinase activity 119. cGMP-dependent protein kinase (PKG), a redox sensor activated by H_2_O_2_-dependent oxidation of Cys-42, is involved in MAPK activation 120, 121. In addition to influencing the upstream of MAPKs, ROS can also activate MAPKs by directly inhibiting MAPK phosphatases. JNK-inactivating phosphatases have been shown to be inhibited by ROS through reversible oxidation of a catalytic site cysteine to sulfenic acid, thus sustaining JNK activation 122.

#### ROS promote EMT

Epithelial-to-mesenchymal transition (EMT) has been defined as an early event in cancer metastasis, which is linked with loss of cell-to-cell adhesion, loss of interaction with the extracellular matrix (ECM) and migration towards blood and lymphatic vessels 123. ROS are involved in these processes by inducing Rho family GTPase-dependent cytoskeletal rearrangement, promoting matrix metalloprotease (MMP)-dependent ECM protein degradation and accelerating hypoxia-inducible factor (HIF)-dependent angiogenesis 124, 125 (Figure 2).

EMT is initiated by loss of cell polarity and detachment from surrounding cells via a complex cascade of cytoskeletal rearrangement brought about by the coordinated action of small Rho family GTPases 126. Among Rho family GTPases, RAC1 promotes membrane protrusion into lamellipodia and filopodia and the establishment of focal contacts at the leading edge 127. An intriguing and obvious question is whether a potential relationship exists between the effects of RAC1 on cytoskeletal dynamics and its capacity for ROS generation by participating in NOX complex assembly. Along this line of investigation, Moldovan et al. (1999) showed that actin cytoskeleton reorganization induced by RAC1 in human endothelial cells required the production of superoxide 128. Furthermore, the transient increase in ROS levels induced by RAC1 activation causes fibroblast cell adhesion and spreading onto fibronectin by upregulating focal adhesion kinase (FAK) expression through reversible oxidation of low molecular weight PTP (LMW-PTP) 129. RAC1 and RhoA (Rho family GTPase A) appear to orchestrate two different and mutually exclusive motility programs in invading malignant cells. In an attempt to clarify the mechanism by which RAC1 regulates RhoA signaling, Nimnual et al. (2003) found that ROS derived from RAC1 inactivated LMW-PTP, causing hyperphosphorylation of p190Rho-GTPase activating protein (GAP), thereby inhibiting RhoA expression 130. Collectively, these observations clearly indicate that RAC1 is an important node in the crosstalk between oxidative stress and cytoskeletal rearrangement.

The critical stage in EMT is scavenging the surrounding physical obstacles. MMPs compose a family of endopeptidases transactivated by nuclear factor-κB (NF-κB), which can cleave almost every protein component of the ECM 131. It has been reported that RAC1-dependent ROS production regulates the expressions of MMPs. In human articular chondrocytes, stimulation of integrin-α5β1 by fragments of the ECM protein fibronectin increases intracellular levels of ROS and leads to increased MMP-13 expression by activating NF-κB phosphorylation 132. The antioxidant agent NAC can completely block this regulatory cascade. Epidermal growth factor (EGF) enhances the invasive capacity of PANC-1 human pancreatic cancer cells by inducing secretion of the collagenase MMP-2. The signaling events downstream of the EGF receptor involve RAC1-generated ROS, which are responsible for NF-κB activation and MMP-2 secretion 133. Taken together, these evidences show that MMPs can be regulated by integrin-ECM-interacting signaling pathways that are susceptible to modification by ROS.

Emerging lines of evidences indicate that hypoxia-induced factors (HIFs) are frequently active key transcriptional regulators which orchestrate signal transduction cascades to induce angiogenesis 134. Major HIF isoforms are accumulated in hypoxic condition and are rapidly degraded in the presence of oxygen due to prolyl hydroxylation by prolyl hydroxylases (PHDs) and subsequent ubiquitin-mediated degradation by Von Hippel Lindau (VHL) 135. Compelling lines of evidences indicate that HIFs are involved in redox regulation via multiple mechanisms. Mild oxidative stress promotes p300 de-SUMOylation by retarding the degradation of Sentrin/SUMO-specific proteases (SENPs) and subsequently enhances the binding of p300 to HIF-1α 136. In addition, hydrogen peroxide inhibits prolyl hydroxylation to promote HIF stabilization by oxidizing catalytic ferrous iron in PHD and inhibiting its activity, and this effect can be reversed by the antioxidant agent vitamin C 137. Moreover, it has been recently reported that NAC exerts antitumoral effects in tumorigenic mouse models mainly by decreasing HIF-1α expression 138.

### The tumor-suppressive roles of ROS

**Figure 3:**
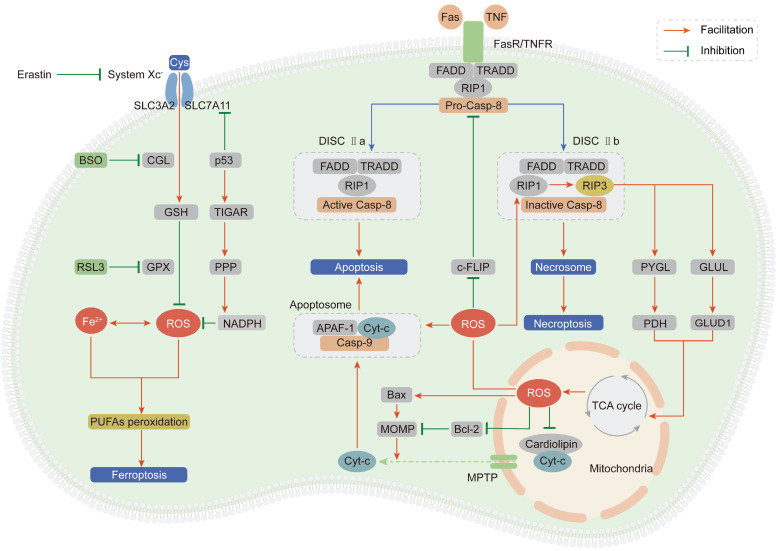
** ROS induce cell death, mainly including apoptosis, necroptosis and ferroptosis.** (Ⅰ) Exceeding ROS promote both the extrinsic and intrinsic apoptosis pathway: ROS activate extrinsic apoptosis pathway by accelerating the ubiquitin degradation of c-FLIP, then enhancing the binding between the adaptor protein and pro-caspase-8; ROS induce intrinsic apoptosis by facilitating the release of Cyt-c from mitochondria to cytoplasm to form apoptosome with casp-9 and APAF-1. (Ⅱ) ROS and necroptosis form a positive feedback loop: ROS stabilize RIP3 protein to lead to the formation of DISC Ⅱb (necrosome); in turn, RIP3 can facilitate the TCA cycle and aerobic respiration in mitochondria to induce ROS generation. (Ⅲ) Ferroptosis is a ROS-dependent form of RCD: the basic of ferroptosis is GSH anabolism disorder leads to the lethal accumulation of PUFAs peroxidation; p53 plays opposite roles on ROS and ferroptosis by inhibiting SLC7A11 expression or increasing NADPH production.

As outlined above, ROS comprise multiple signaling entities with opposite effects and diverse spatiotemporal functions in cancer progression. When the ROS accumulation exceeds the tipping point, their carcinogenic roles in proliferation and invasion are shifted to antitumor effects via the induction of regulated cell death (RCD) programs, mainly including apoptosis, necroptosis and ferroptosis (Figure 3).

#### Apoptosis

Apoptosis, also known as type I programmed cell death, is mediated by extrinsic and intrinsic pathways, which are executed by specific cysteine proteases known as caspases 139.

The extrinsic apoptotic pathway is driven by the ligand-receptor interaction between death-inducing ligands such as Fas ligand (FasL) and tumor necrosis factor (TNF) and their respective receptors Fas receptor (FasR) and TNF receptor (TNFR) 140, 141. After the ligand-receptor interaction, an adaptor protein (FADD for FasR and TRADD for TNFR), receptor-interacting protein kinase 1 (RIP1) and procaspase-8 are recruited to form the death-inducing signaling complex (DISC) and subsequently induce apoptosis 142. ROS stimulate the extrinsic apoptotic pathway by accelerating the ubiquitin-mediated proteasomal degradation of the antiapoptotic factor cellular FLICE-inhibitory protein (c-FLIP), which impedes formation of the DISC through competitive binding with procaspase-8 for the adaptor protein 143, 144. Pretreatment with NAC can effectively stabilize the c-FLIP protein and facilitate apoptosis, demonstrating that ROS are apoptosis-inducing factors.

The intrinsic apoptotic pathway is activated in a mitochondria-dependent manner by release of the proapoptotic factor cytochrome-c (Cyt-c) from mitochondria through the mitochondrial permeability transition pore (MPTP) 145. In mitochondria, Cyt-c is immobilized by reduced cardiolipin. After cardiolipin is oxidized by ROS derived from ETC, its affinity for Cyt-c is attenuated, leading to Cyt-c penetration and release into the cytosol 146. Then, Cyt-c interacts with apoptotic protease activating factor 1 (APAF-1) and procaspase-9 to form the apoptosome, subsequently activating the caspase-9 signaling cascade and inducing apoptosis 147. Enoksson et al. (2005) showed that overexpression of glutaredoxin 2 (GRX2) in HeLa cells specifically inhibited Cyt-c release and caspase activation by preventing cardiolipin oxidation in mitochondria 148. In addition to Cyt-c, caspase-9 is a direct target of ROS. Zuo et al. (2009) demonstrated that oxidative modification of Cys-403 in caspase-9 promoted a disulfide-mediated interaction with APAF-1 and facilitated autocleavage-mediated activation of caspase-9 149. The last but not the least, ROS accumulation enhances mitochondrial outer membrane permeabilization (MOMP) via regulating B-cell lymphoma-2 (Bcl-2) family and subsequently induces the release of Cyt-c 150, 151. The ratio between two hostile proteins of BCL-2 family (pro-apoptotic Bax vs anti-apoptotic Bcl-2) is modulated by ROS via following mechanisms: direct oxidation of Bcl-2 at Cys-158 and Cys-229 152, decreasing ubiquitination of Bax and increasing ubiquitination of Bcl-2 153, 154.

#### Necroptosis

Necroptosis, termed type III programmed cell death, is initiated in a manner similar to the extrinsic apoptotic pathway: the ligand-receptor interaction between TNFR or FasR and their respective ligands 155. However, necroptosis is caspase-independent and involves receptor-interacting protein kinase 3 (RIP3) to form DISC IIb, distinct from DISC IIa in apoptosis 156. Accumulating evidences suggest that necroptosis plays a vital role in cancer biology and influences the prognosis of patients who receive antineoplastic chemotherapy or radiotherapy 157. Inducing necroptosis has emerged as a novel approach for bypassing apoptosis-resistance and a new target for cancer therapy.

Based on a series of studies, we draw the conclusion that ROS and necroptosis can form a positive feedback loop. Excessive ROS oxidize the Cys-257, Cys-268 and Cys-586 residues of RIP1 and subsequently activate RIP1 through Ser-161 autophosphorylation, which protects the RIP3 protein from cleavage by caspase-8 and leads to the formation of the DISC IIb 158. In turn, RIP3 can facilitate the TCA cycle and aerobic respiration in mitochondria to induce ROS generation through two distinct metabolic signaling pathways: (1) upregulation of glycogen phosphorylase (PYGL) and pyruvate dehydrogenase (PDH) expression 159, 160; (2) elevation of glutamate-ammonia ligase (GLUL) and glutamate dehydrogenase 1 (GLUD1) expression to increase glutaminolysis 161.

#### Ferroptosis

Ferroptosis is an iron- and ROS-dependent form of regulated cell death (RCD), and is morphologically, biochemically, and genetically distinct from apoptosis and necroptosis 162. The oxidative stress burden caused by an excessive intracellular iron level and inadequate GSH leads to lethal accumulation of peroxidated polyunsaturated fatty acids (PUFAs), which is the fundamental characteristic of ferroptosis 163. Recently, growing researches suggest that small molecules-induced ferroptosis has a strong inhibition of tumor growth and enhances the sensitivity of chemotherapeutic drugs, especially in the condition of drug resistance 164. These evidences have highlighted the importance of ferroptosis in cancer therapy.

In 2012, Dixon et al. first proposed the concept of ferroptosis by using the oncogenic RAS-selective lethal (RSL) small molecule erastin to trigger an iron- and ROS-dependent form of cell death. This group unequivocally proved that erastin can inhibit cystine uptake through the cystine/glutamate antiporter (system X_C_^-^) and impair the GSH-dependent GPX antioxidant system, ultimately leading to ferroptosis 165. Cotreatment with the iron chelator deferoxamine or the lipid ROS scavenger ferrostatin-1 can significantly reverse the lethal effect of erastin. In addition to erastin, RAS-selective lethal compound 3 (RSL3) can also lead to ferroptosis, not by blocking system X_C_^-^ but by inhibiting GPX4, which can be counteracted by GPX4 overexpression 166. Moreover, buthionine sulfoximine (BSO), a GSH deletion agent, has been proven to initiate ferroptosis by inhibiting the activity of glutamate-cysteine ligase (GCL), which is the rate-limiting enzyme in GSH synthesis 167.

System X_C_^-^ is a dimeric channel protein that is structurally composed of SLC7A11 and SLC3A2 and is responsible for maintaining redox homeostasis by transporting cystine to synthesize GSH 168. Recently, it was demonstrated that p53 can inhibit cystine uptake by repressing SLC7A11 expression and inducing ferroptosis 169. Combining these findings with the abovementioned ROS-dependent carcinogenic roles in Trp53-knockout mice, we summarize strong evidences to support the idea that p53 plays paradoxical roles to dynamically maintain ROS balance. On one hand, p53 induces TIGAR expression to redirect glucose towards metabolism through the PPP and increase cytosolic NADPH production 170. On the other hand, p53 impedes cystine uptake through system X_C_^-^ to weaken the antioxidant defense system 169.

## The application of antioxidants in cancer prevention

ROS accumulation in normal cells is one of the initiating factors in the early stage of the neoplastic process. Therefore, an appropriate application of antioxidants can decrease the oxidative stress burden, consequently preventing normal cells from sliding into the abyss of malignant transformation. Numerous epidemiologic data and preclinical/clinical studies have suggested that keeping an antioxidative dietary or pharmaceutical application of antioxidative phytochemicals can effectively prevent tumorigenesis.

### An antioxidative dietary reduces cancer incidence

It is widely accepted that fruit and vegetables rich in antioxidant nutrients are important components of a healthy diet and can reduce the incidence of numerous malignancies. In a study with 77,446 participants, Han et al. (2013) measured the uptake of different antioxidants from the diet in relation to pancreatic cancer risk. They observed that dietary selenium intake was negatively associated with the incidence of pancreatic cancer 171. In another cohort study, Wright et al. (2004) constructed a dietary antioxidant index that analyzed the comprehensive intake of individual selenium, flavonoids, vitamin C and carotenoids to predict the risk of lung cancer 172. This group proved that integration of dietary antioxidants can significantly reduce lung cancer incidence in male smokers. In animal models, administration of tomato powder, which is rich in the antioxidant carotenoid lycopene, reduces steatosis and inflammatory foci and abolishes preneoplastic foci in liver tissues of mice injected with diethylnitrosamine (DEN) 173. Cocoa, with abundant antioxidant properties, can decrease malondialdehyde (MDA) levels, which were elevated in a mouse model of azoxymethane (AOM)/dextran sulfate sodium (DSS)-induced colitis-associated colorectal cancer. Further investigations showed that cocoa treatment could upregulate cellular enzymatic antioxidants, such as SOD, CAT, GPX and GR, by activating the NRF2 signaling cascade 174. In addition, it has been shown that consumption of ≥150 g of tea per month can effectively protect women against esophageal carcinoma 175.

### Application of antioxidative phytochemicals prevents tumorigenesis

Phytochemicals, secondary plant metabolites with antioxidant properties, play important roles in cancer chemoprevention by reversing oxidative stress-induced malignant transformation. Detoxification of ROS via positively regulating phase II antioxidant enzymes is a main factor contributing to the chemopreventive potential of phytochemicals 176.

Curcumin, contained in turmeric has been demonstrated to have preventive effects against multiple types of chemical-induced spontaneous neoplasms in animal models by inducing the expression of phase II antioxidant enzymes through activating the KEAP1/NRF2/AME pathway 177, 178. Epigallocatechin gallate (EGCG), a key active catechin in green tea known to possess antioxidant capacity, has been shown to slow the formation of aberrant colon crypt foci induced by 2-amino-3-methylimidazo [4,5-f] quinoline via activation of the NRF2 signaling pathway 179. Moreover, in a one-year proof-of-principle clinical study, Bettuzzi et al. (2006) proved that green tea catechins significantly decreased total prostate-specific antigen levels and delayed the emergence of prostate cancer in volunteers with high-grade prostate intraepithelial neoplasia 180.

In recent years, great efforts have been made to investigate the roles of phytochemicals in cancer prevention. Owing to space constraints, these relevant outstanding achievements are summarized in Table 1.

## The roles of pro-oxidants in cancer therapy

It is widely recognized that the antitumor effects of pro-oxidants are attributed to the induction of excessive oxidative stress and subsequent ROS-dependent cell death, which can be achieved by ROS-induction or antioxidant-inhibition therapies. As summarized in Table 2, multiple drugs with direct or indirect effects on ROS accumulation have been used clinically for cancer treatment. A more detailed investigation of the influences of these drugs on redox metabolism would be informative for designing individualized treatments with fewer side effects and a lower propensity of drug resistance.

### ROS-inducing therapies

Traditional antineoplastic therapies, including chemotherapy and radiotherapy, are currently used to induce excessive levels of oxidative stress to selectively kill cancer cells. Patients who receive these treatments exhibit signs of ROS-induced cell death, DNA damage and lipid peroxidation.

#### Chemotherapy

Conventional chemotherapeutics such as anthracyclines and platinum coordination complexes generate extremely high levels of ROS 181. For example, doxorubicin (DOX), an anthracycline antibiotic, induces oxidative DNA damage and caspase-dependent apoptosis initiated by direct H_2_O_2_ generation through NOX activation in human leukemia cells 182. Moreover, in p53-null human osteosarcoma Saos-2 cells, ROS levels were increased, with subsequent mitochondrial membrane depolarization and Cyt-c release after 48 h of DOX treatment, and CAT abolished the proapoptotic effects of DOX 183. In addition, it was found that DOX was indiscriminatingly localized at the mitochondria of normal cells and competes with coenzyme Q_10_ in ETC to induce ROS production, which is the basis of its cardiotoxicity 184. Cisplatin, another classical conventional anticancer drug recognized as an agent that induces a mitochondrial-dependent ROS response, which significantly enhances the cytotoxic effect caused by mitochondrial DNA damage 185. In HCT116 colon carcinoma cells, the apoptotic activity of cisplatin is dependent on p53-associated ROS accumulation and downstream p38 MAPK activation 186. Treatment with antioxidants (ascorbic acid and dehydroascorbic acid) or a p53 inhibitor (pifithrin-α) can block cisplatin-induced apoptosis and reduce the generation of ROS. Similar to DOX, oxidative stress exacerbation by cisplatin occurs not only in cancer cells but also in normal cells. For instance, hearing loss, one of the common side effects caused by the ototoxicity of cisplatin, is induced by excessive generation of ROS in cochlear cells and can be effectively prevented or alleviated by the application of antioxidants 187. In addition to DOX and cisplatin, 2-methoxyestradiol (2ME2), a metabolite of estradiol-17 beta, is also known to induce apoptosis in tumor cells via ROS generation. Mechanistically, 2ME2 inhibits mitochondrial respiration by inactivating complex I and shunts electrons leaked from ETC to form superoxide, subsequently resulting in Cyt-c release and caspase-9 activation 188.

#### Radiotherapy

Early in the 1950s, it was indeed well known that a high local concentration of molecular oxygen enhanced the efficacy of radiotherapy, as DNA lesions caused by ROS generated during H_2_O radiolysis can react with O_2_ to form superoxide anions 189. Recent studies have shown that endogenous ROS, whether generated by the mitochondrial ETC or NADPH oxidases, can be activated by radiation exposure, leading to persistent oxidative stress in tumor cells 190, 191. Because the ROS level is the critical mediator of irradiation-induced cell death, the mechanisms of radiation resistance are associated with impaired ROS production or a powerful antioxidant system. It has been shown that pharmacologic depletion of GSH by BSO in cancer stem cells (CSCs) significantly decreases their clonogenicity and results in radiosensitization 192. Specifically, increasing ROS levels in tumor cells during radiotherapy can significantly enhance the efficiency and decrease the dosage of radiation, subsequently reducing nonselective killing of normal cells and severe systemic side effects on bystander organs. With this insight, Chen et al. (2019) developed a Gd-doped titania nanosensitizer that targets mitochondria to achieve efficient radiotherapy by triggering a "domino effect" of ROS accumulation, mitochondrial permeability transition, Cyt-c release and caspase-dependent cell apoptosis 193.

### Antioxidant-inhibiting therapies

GSH and TRX are central players in antioxidant systems by transmitting reducing equivalents from NADPH to oxidized molecules through PRX- and GPX-dependent intracellular enzymatic redox reactions. Thus, antineoplastic therapeutic strategies selectively targeting GSH and TRX metabolism in cancer cells are effective measures to enhance the potency of ROS modulation.

#### 6.2.1 Drugs that affect GSH metabolism

Compared to normal cells, cancer cells with high GSH content seem to be more sensitive to a selective GSH depletion strategy. As noted above, GCL is the rate-limiting enzyme in GSH synthesis and has been considered as an antineoplastic target for over 30 years 194. Buthionine sulfoximine (BSO), an irreversible inhibitor of GCL, is most frequently used as an adjunct to synergize with other chemotherapies. Gana et al. (2019) proved that multidrug resistance protein 1 (MRP1) modulators (verapamil and apigenin) synergize with BSO to collaterally sensitize MRP1-expressing cancer cells to arsenic trioxide and selectively kill them 195. In high-risk neuroblastoma, increased cellular GSH induces resistance to melphalan, a myeloablative drug. In 2016, a phase I trial of BSO and melphalan with autologous stem cells for recurrent/refractory neuroblastoma was conducted by Villablanca et al. The results showed that a combination of BSO plus melphalan was feasible and effective in the treatment of neuroblastoma 196. Moreover, in a preclinical animal trial, the cytotoxic effect of azathioprine plus BSO was found to be effective for localized treatment of hepatocellular carcinoma, and further *in vitro* studies suggested that GSH depletion by BSO gave rise to Cyt-c release and mitochondrial-dependent cell death 197.

Cystine imported into the cytosol by system Xc^-^ is an important material for GSH synthesis. Sulfasalazine, an anti-inflammatory drug used for various types of arthritis 198, has been found to specifically inhibit system Xc^-^ activity 199. Treatment with sulfasalazine led to reduced uptake of cystine and subsequent depletion of GSH, which inhibited the proliferation of human pancreatic cancer cells both *in vitro* and *in vivo* 200. In human small cell lung cancer cells, it was proven that sulfasalazine was potentially useful as a target for therapies based on GSH depletion 201.

#### 6.2.2 Drugs that affect TRX metabolism

As mentioned above, TRX, the molecular substrate of peroxiredoxin (PRX), participates in the scavenging of H_2_O_2_ to maintain redox balance. In addition, the reduced form of TRX-(SH)_2_ can directly reduce disulfide groups in oxidized proteins, and the oxidized form of TRX-(S)_2_ is then reduced by thioredoxin reductase (TRXR) in an NADPH-dependent manner. In recent years, TRXR/TRX has been recognized as an important modulator of tumor development; hence, targeting TRXR/TRX is a promising strategy for antitumor therapy. Auranofin, a gold compound initially utilized for the treatment of rheumatoid arthritis 202, is a strong inhibitor of TRXR in both the cytosol and mitochondria 203. In 2014, Fiskus et al. reported the identification of auranofin in repurposing for the treatment of chronic lymphocytic leukemia (CLL) by inhibiting TRXR activity and increasing intracellular ROS levels 204. Similarly, Pessetto et al. (2013) attempted to reposition auranofin to treat metastatic gastrointestinal stromal tumor (GIST). They found that auranofin dramatically inhibited GIST cell growth and suggested the clinical benefit of auranofin in GIST patients, particularly those with imatinib resistance 205. Ethaselen (1,2-[bis(1,2-benzisoselenazolone-3(2H)-ketone)]ethane, BBSKE), is a novel TRXR-targeted organoselenium compound and is currently under investigation in clinical trial NCT02166242 for the treatment of advanced non-small cell lung cancer (NSCLC) 206. In previous studies, ethaselen has shown antineoplastic effects against prostate, lung, colon and gastric cancer both *in vivo* and *in vitro* by suppressing TRXR activity, and elevating ROS levels, and subsequently inducing cell death 207-210.

PX-12 (1-methylpropyl 2-mercaptoimidazolyl disulfide), also called IV-2 in early references, was initially identified as a competitive inhibitor of TRXR-mediated reduction of TRX in 1998 211. Recently, PX-12 has been proven to inhibit the growth and induce the apoptosis of lung, cervical, liver, gastric and colorectal cancer cells by decreasing reduced TRX expression and increasing ROS levels 212-216. Although PX-12 did not appear to be clinical efficacy in a randomized phase II study in pancreatic cancer, its potential as a clinical anticancer candidate is still promising 217.

## Future perspectives

A major question in the field of cancer redox biology is whether ROS can function as specific weapons to destroy tumor cells and not in the dualistic role, indiscriminately damaging normal cells. This question is the basis of many controversies in the field of redox biology and accounts for the conflicting results of clinical trials and experimental studies. Although measures to globally elevate ROS to cytotoxic levels have promising availability for killing cancer cells, such strategies inevitably induce systemic toxicity much like conventional chemo- and radiotherapeutic regimens. Leveraging ROS modulation for the development of safe and efficient anticancer therapies necessitates experimental delineation of the distinct redox signaling pathways that exclusively contribute to cancer cell growth and survival. In this regard, further elucidation of ROS-related cysteine modifications and their functional consequences will be fundamental to advancing our understanding of the selective effects of ROS on cancer and normal cells. New molecular probes that allow monitoring of ROS with temporal and spatial specificity will shed further light on the complicated regulatory relationship between different redox couples and their downstream influences on different subcellular organelles. In spite of scientific quandaries and technical challenges, the prospect of interdisciplinary collaborations between oncologists, pharmacists and biochemists towards a better understanding of cancer-specific redox signaling events holds promise for overcoming the controversial side effects of pro-oxidant antineoplastic therapies.

## Figures and Tables

**Table 1:** Antioxidative phytochemicals in cancer prevention.

Phytochemicals	Cell line/tumor model	Anti-cancer effects
Curcumin	Pancreatic cancer cells (BxPC3 and PANC1)	Suppresses cancer cell migration and invasion 218
	Breast cancer cells (MDA-MB-231, BT-483, and MCF7)	Suppresses cancer cell proliferation and invasion 219, 220
	NSCLC cell line (A549 and H460)	Decreases *in vitro* metastatic progression and increased apoptosis 221, 222
	Prostate cancer cells (PC12)	Inhibits cancer cell invasion 223
	UV-induced skin injury model	Reduces UV-induced cytotoxicity 224
	Dalton's lymphoma bearing mice	Reduces tumor invasiveness 225
	BaP-induced forestomach tumorigenesis	Reduces tumor growth 226
EGCG	Mammary epithelial cells (MCF10A)	Enhances antioxidant defense capacity 227
	Liver cancer cells (HepG2)	Reduces exogenous oxidative stress 228
	Orthotropic mouse model of colon cancer	Reduces primary tumor growth and its metastasis to liver and lungs 229
	Carcinogen-induced mouse model of colon carcinogenesis	Protective role against colon carcinogenesis 179
	Immortalized human keratinocyte (HaCaT)	Protects skin against ionizing-radiation against DNA damage 230
Resveratrol	Mammary epithelial cells (MCF10A)	Protects against (4-OHE2)- induced migration and transformation 231
	Pancreatic cancer cells (BxPC3 and PANC1)	Inhibits ROS-induced proliferation and migration 232
	Prostate cancer cells	Inhibits DHT-induced progression 233
	Estrogen induced breast carcinogenesis	Protects from oxidative stress and its associated DNA damage 234, 235
	Rat model of hepatocarcinogenesis	Suppresses oxidative stress 236
Hesperidin	Azoxymethane induced Liver carcinogenesis	Inhibits burden of Hepatic tumors 237
	NSCLC cell line (A549)	Suppresses migration and invasion *in vitro* 238
2'-hydroxyflavanone	Renal cancer	Inhibits survival of cancer cells *in vitro* and tumors *in vivo* 239, 240
	Lung cancer cell lines	Inhibits growth in nude mouse xenograft models 241
	Breast cancer	Inhibits cancer cell survival, cell cycle *in vitro* and tumor progression *in vivo* 242
Quercetin	Lung cancer	Suppresses metastasis 243
	Breast cancer	Incudes apoptosis and necroptosis 244
	Liver cancer	Inhibits proliferation and induces apoptosis 245

**Table 2:** Pro-oxidative drugs in antineoplastic therapies.

Drug	Pharmacological mechanism	Cancer types	Phase (status)	Clinical trial ID
**Directly affect redox metabolism**				
NOV-002	Glutathione disulphide mimetic; alters intracellular GSSG/GSH ratio 246	Non-Small Cell Lung Carcinoma	Phase Ⅲ (Completed)	NCT00347412
		Ovarian Cancer	Phase Ⅱ (Completed)	NCT00345540
		Leukemias / Myelodysplastic Syndrome	Phase Ⅱ (Withdrawn)	NCT00960726
L-asparaginase	Depletes glutamine; reduces GSH 247	Acute Lymphoblastic Leukaemias (ALL)	Phase Ⅳ (Completed)	NCT00494897
		Peripheral T Cell Lymphoma (PTCL)	Phase Ⅳ (Recruiting)	NCT03071822
		Acute Myeloid Leukemia (AML)	Phase Ⅲ (Recruiting)	NCT04293562
		Adenocarcinoma of the Pancreas	Phase Ⅲ (Recruiting)	NCT03665441
Sulphasalazine	Inhibitor of system XC-; reduces intracellular transport of cystine required for GSH synthesis 200	Glioblastomas	Phase Ⅰ (Recruiting)	NCT04205357
Buthionine sulphoximine (BSO)	Glutamate-cysteine ligase complex inhibitor; inhibits *de novo* GSH synthesis 195	Neuroblastomas	Phase Ⅰ (Completed)	NCT00002730
		Melanoma	Phase Ⅰ (Withdrawn)	NCT00661336
Arsenic trioxide (As_2_O_3_)	Reacts with cysteine residues on crucial proteins; inhibits mitochondrial respiratory function, thereby increasing ROS generation 248	Acute Promyelocytic Leukemia (APL)	Phase Ⅳ (Active Not Recruiting)	NCT01987297
		Neuroblastoma	Phase Ⅱ (Completed)	NCT00024258
		Small Cell Lung Cancer (SCLC)	Phase Ⅱ (Completed)	NCT01470248
		Cervical Cancers	Phase Ⅱ (Completed)	NCT00005999
		Liver Cancer	Phase Ⅱ (Completed)	NCT00128596
**Indirectly affect redox metabolism**				
Celecoxib	A selective cyclooxygenase-2 (COX-2) inhibitor; induction of ROS by inhibiting mitochondrial oxygen consumption 249	Colorectal Cancers	Phase Ⅳ (Completed)	NCT00473980
		Hepatocellular Carcinoma (HCC)	Phase Ⅳ (Completed)	NCT02961998
		Biliary-pancreas Cancer	Phase Ⅳ (Active Not Recruiting)	NCT01111591
		Breast Cancer	Phase Ⅲ (Completed)	NCT02429427
		Lung Cancers	Phase Ⅲ (Terminated)	NCT01041781
Nelfinavir	Originally developed as HIV protease inhibitor but it also induces mitochondrial ROS production 250	Cervical cancer	Phase Ⅲ (Recruiting)	NCT03256916
		Adenoid Cystic Cancer of the Head and Neck	Phase Ⅱ (Completed)	NCT01065844
		AIDS-Related Kaposi's Sarcoma	Phase Ⅱ (Completed)	NCT00003008
		NSCLC	Phase Ⅱ (Terminated)	NCT01108666 NCT00791336
Bortezomib	Proteasome inhibitor; induces ROS owing to mitochondrial dysregulation and ER stress 251	Multiple Myeloma	Phase Ⅳ (Completed)	NCT02268890
		Acute Myeloid Leukemia (AML)	Phase Ⅲ (Active Not Recruiting)	NCT01371981
Anthracyclines (doxorubicin)	Induce the generation of ROS through two main pathways: a non-enzymatic pathway that utilizes iron, and an enzymatic mechanism that involves the mitochondrial respiratory chain 252	Follicular Lymphoma	Phase Ⅳ (Active Not Recruiting)	NCT03817853
		Breast Cancer	Phase Ⅳ (Active Not Recruiting)	NCT02419742
		Multiple Myeloma	Phase Ⅳ (Active Not Recruiting)	NCT02577783
		Non-Hodgkin's Lymphoma (NHL)	Phase Ⅳ (Completed)	NCT00969462
		HCC	Phase Ⅲ (Active Not Recruiting)	NCT01015833
2-methoxyestradiol	Metabolite of estradiol-17β; induces ROS through the loss of mitochondrial membrane potential 253	Renal Cell Carcinoma	Phase Ⅱ (Completed)	NCT00444314
		Ovarian Cancer	Phase Ⅱ (Completed)	NCT00400348
		Prostate Cancer	Phase Ⅱ (Completed)	NCT00394810
Fenretinide (4-hydroxyphenyl retinamide)	Synthetic retinoid derivative; induces apoptosis through the production of ROS and mitochondrial disruption 254	Breast Cancer	Phase Ⅲ (Completed)	NCT00002646
		Bladder Cancer	Phase Ⅲ (Completed)	NCT00004154
		Prostate Cancer	Phase Ⅱ (Completed)	NCT00080899
		Head and Neck Carcinoma	Phase Ⅱ (Completed)	NCT00006471
		Lung Cancers	Phase Ⅱ (Completed)	NCT00009971
		Renal Cancers	Phase Ⅱ (Completed)	NCT00011973
